# On the use of surveys and interviews in social studies of cancer: understanding incoherence

**DOI:** 10.3332/ecancer.2019.918

**Published:** 2019-03-28

**Authors:** Carlo Caduff, Pooja Sharma, CS Pramesh

**Affiliations:** 1 Department of Global Health & Social Medicine, King’s College London, 30 Aldwych, London WC2B 4BG, UK; 2 Faculty of Social Sciences, Jamia Milia Islamia, New Delhi 110025, India; 3 Tata Memorial Centre, Dr Ernest Borges Road, Mumbai 400012, India

**Keywords:** methodology, surveys, interviews, data collection, data analysis, incoherence

## Abstract

In this article, we discuss the advantages and disadvantages of the survey as a methodological tool in social studies of cancer. Drawing on our own research on the accessibility and affordability of cancer care in India, we present examples from interviews and identify some limitations inherent in survey-based research approaches. We argue that social studies of cancer require a more rigorous methodology to produce robust and reliable data.

## Introduction

In an editorial comment published in the European Journal of Cancer, David Weller emphasised the importance of understanding the social aspects of cancer care ‘at all stages of the journey, from early detection and screening to the active treatment stage and late palliation. At all stages, the message is clear; adequate attention must be given to psychosocial and behavioural factors if we are to develop cancer strategies which are truly focused on individuals with cancer, their families and carers’ [1]. Referring to a report published by an expert group funded by the European Union, Weller noted that there is a lack of systematic social science research on cancer.

In the report discussed by Weller, the expert group outlined a number of social science research needs in cancer prevention, cancer screening and cancer treatment [2]. It called for research capacity building and suggested the development of new research methodologies. In this article, we argue that social studies of cancer do not necessarily require novel methodologies. The tool kit of social science research is already quite rich. What we find more urgent is to rethink the preference for one particular method: the survey. The majority of social studies of cancer in oncology are based on surveys. Quantitative methodologies with large samples are favoured over qualitative methodologies with smaller samples.

The survey has become the preferred tool for large sample size data collection in cancer research because of its simplicity. It is easy to design and deploy. In this type of research, respondents are requested to provide information either by structured interview or by questionnaire [3–7]. Focusing on the generation of standardised information, surveys provide data that can be quantified. Compared with semi-structured interviews that use open-ended questions, the advantages are significant and obvious. A single semi-structured interview can take between 30 and 90 minutes. It needs to be transcribed and translated. Once it has been transcribed and translated, the narratives (which can be confusing and contradictory) need to be analysed. This is a very time-consuming process involving multiple analytical steps.

The emphasis on large samples makes it more difficult for researchers to engage in alternative research designs. To conduct 400 or 500 semi-structured interviews with open-ended questions is extremely time-consuming and produces an enormous amount of information that is not easily quantifiable. This makes the survey the preferred tool in oncology for the collection of standardised information on social aspects of cancer. Given the constraints of time and money, it comes as no surprise that the survey has become the dominant method in social studies of cancer.

However, it is well known that survey-based data are not always robust and reliable. In this article, we argue that cancer, a cluster of diseases often set apart from other diseases, poses important challenges. In the following section, we present examples from semi-structured interviews that highlight some of the issues that social science research faces when studying cancer.

These examples are drawn from a Wellcome Trust funded social science project on cancer care in India. The project is based on 400 semi-structured interviews with patients and family members. In these interviews, we asked open-ended questions focused on the accessibility and affordability of cancer care in India. All interviews were conducted in Tata Memorial Hospital in Mumbai between October 2017 and April 2018. For the research, we received approval from the Institutional Ethics Committees of Tata Memorial Hospital, in addition to approval by the Indian Council of Medical Research of the Government of India. The interviews were conducted in Hindi, Bengali, Marathi and English, depending on the preferred language of participants. We are currently in the process of translating and analysing all interviews. What we present in this article are not the overall results of the study. Rather, our aim is to discuss some methodological issues that we encountered while doing semi-structured interviews with patients and family members. In this article, our examples focus on one question in particular: We asked participants whether there had been other cases of cancer in the family or neighbourhood or village. As we will see, cases of cancer often appear in accounts in oblique ways. This has important implications, especially when compared with survey-based studies.

## Hearing about cancer: three examples

### Example 1

This excerpt comes from an interview with a breast cancer patient who is undergoing treatment at Tata Memorial Hospital in Mumbai. The entire interview is 60 minutes long and was conducted in Hindi.

Q: You have never done self-examinations?

A: All these things didn’t occur to me at the time. Now, the doctor has said that I have to examine every month. At that time I didn’t know. Now, I have told all my sisters to keep doing this. Now that I have come to know I will do it. Like I said, there was no family history. So, I didn’t know anything. The word cancer has entered our life only now.

**Same interview with the same person after 30 minutes and questions about other topics**

Q: Have doctors asked you about cases of cancer in your family?

A: Yes. My father has it. But that is of a different type. Even the doctors couldn’t understand it, that’s why I say he doesn’t have it.

Q: Your father? Where did he have it?

A: He has it in the blood or the bladder; the doctors are also not able to understand it. He said that after a certain age, it happens, and it’s in an early stage. So, we also haven’t understood the problem. He is still around and is quite healthy. He has to take some medicine and he will remain fine with that.

Q: What type of medicine is it?

A: I don’t know.

Q: You don’t know?

A: I don’t know the name. My brother looks after all this. He knows it. He has to take it lifetime until he is around. He is 70 years old and his diet is good and he is quite healthy. So, we don’t think that he has something.

Q: That’s why you didn’t say it to the doctor just before when he asked you about cancer in the family…?

A: I didn’t say anything. If I had mentioned it, then they would have asked: what is it? So what would I have said? Blood cancer or what cancer should I say? What should I explain to them?

Q: Since when do you know that he has cancer?

A: It has been 12–15 years since we know it.

Q: You have known it for 12–15 years?

A: Well, he is quite normal…

Q: But he received treatment in a cancer hospital?

A: Yes. It’s for cancer only.

### Example 2

This excerpt comes from an interview with a father whose 18-year-old son is undergoing treatment at Tata Memorial Hospital in Mumbai. The entire interview is 20 minutes long and was conducted in Hindi.

Q: Have you ever heard about cancer?

A: No.

***Q:**** You never heard about it in a movie, or on TV, or in the neighbourhood, or in your village?*

A: No, but we knew about TB. We didn’t know about cancer at all. Now, suddenly we have come to know that he [son] has cancer.

Q: Did you ever realise that cancer treatment is so expensive?

A: No, no. How would we know? We came to know about this only when the doctor informed us that day. The doctor said that there would be so much of expenses.

Q: His mother knows that he has cancer?

A: Yes, she knows now. We told her.

Q: What did you tell her? The name of the disease, or that he has been operated and is cured?

A: I told her that it’s cancer.

Q: She understood then. Did she know that cancer is a dangerous disease?

A: How would she know?

Q: She doesn’t know?

A: It has never happened to any one of us, so how would she know?

Q: You never heard anyone around you in your village?

A: My second brother had it. He was operated in Nair Hospital, but he didn’t use to stay with our family and was separate.

Q: Your brother, meaning your cousin or someone?

A: No, no, my real brother.

Q: Your real brother? He had cancer?

A: Yes. Blood. Tumour in the blood.

Q: How many years back?

A: It has been about 10 years now.

Q: Ten years ago?

A: It was in Nair. We didn’t know that Tata also treats cancer, so we went to Nair.

Q: Then he wasn’t cured?

A: No, he wasn’t cured. He passed away.

### Example 3

This excerpt comes from an interview with a farmer from Maharashtra who has come to Tata Memorial with his father, who is the patient. The interview is 30 minutes long and was conducted in Hindi.

Q: Did you ever hear the name of cancer? Or did anyone have it in your house?

A: No, never heard it.

Q: Did you ever hear the name?

A: No, never. First time.

**Same interview with the same person after 20 minutes and questions about other topics**

Q: And [has] this kind of big disease [cancer] [occurred] anywhere in your family or village?

A: No.

Q: What is the kind of perception about this disease in your village?

A: In my village, this disease hasn’t happened to anyone. Not to anyone as yet.

**After 30 minutes and questions about other topics**

Q: You have been here for so many days. You know it’s a cancer hospital; do you ever get scared if you get a pain in your body?

A: Yes. Earlier I used to feel so. In the beginning, when I came, I used to feel so. It’s a cancer hospital. There was a man from my village who brought his son here. He also had cancer. Of the bone. Everything was good for them. They were here for 1.5 years. Recently, they returned to the village. They had all kinds of facilities here. It was a child. They were given all facilities. Now they have left. They said it’s good. You don’t have to take any tension.

**After 35 minutes and other questions about other topics**

Q: Did you hear the name cancer before?

A: Yes. cancer, I had heard the name. My uncle had cancer. He died.

Q: Your real uncle?

A: Yes.

Q: What kind of disease?

A: It was here, in the mouth.

Q: He must have been eating tobacco.

A: He ate tobacco and he also drank alcohol. We took him to Aurangabad. They burnt it [radiation]. Then, we brought him to our village. For 2–4 days, he was fine. Later, he thought that his disease is fine now. He started drinking again. He died.

## Discussion

These three examples show how cancer consistently fails to appear in the initial response to the direct question about other cases of cancer in the family or neighbourhood or village. In the first example, the person didn’t disclose her father’s cancer even to the doctor.

Why are initial responses to questions about cancer often inaccurate? And what are the methodological implications? Equally interesting is how the initial response to the question about cancer changes over the course of the interview. We have seen this happening time and again in our interviews. Initially, research participants tend to respond negatively when asked directly about other cases of cancer in the family or neighbourhood or village. But over the course of the conversation, more and more cases gradually emerge in the narrative. This demonstrates how essential time and patience are from a methodological point of view to establish a relationship and reduce the anxieties of a knowledge test. These anxieties are inevitable because the research design requires participants to reveal their knowledge to someone who is seen as a knowledge expert.

The survey is a tool that tends to have no time for patience: It is designed to be administered quickly. In addition, the survey usually doesn’t rely on redundancy as a research strategy: Typically, a question is asked only once in a survey. The semi-structured interview with open-ended questions, by contrast, takes time and gives time. The conversation can return to the same question, again and again, and approach it from different angles. This makes it possible for different answers to appear. Open-ended questions often trigger stories that coincidentally reveal events that had been forgotten, that had been suppressed, or that seemed irrelevant from the participant’s point of view. As our examples demonstrate, cases of cancer in the family or neighbourhood or village are often expressed indirectly, almost casually, as part of some other story.

It seems clear to us that this difficulty of talking about cancer constitutes a major challenge for the survey with its direct way of questioning and its focus on generating standardisable information. A method that aims to go directly to the point will not be able to capture something that can be stated only indirectly. We believe that cancer, because of its complex mode of existence in people’s lives, is particularly difficult to study from a social science perspective. Information can often be arrived at only indirectly. Accordingly, the most suitable combination of methods promising the most robust and reliable data is a mixed methods approach that includes open-ended questions. Only a mixed methods approach is able to reveal the risk of incomplete and sometimes misleading information emerging from survey data.

## Conclusion

One might wonder why respondents fail to mention family members with cancer when asked about other cases of disease. What accounts for the response? What is the meaning of this difficulty to recognise, recall and report? Is it a form of denial? Does it mean that our interviews have managed to reveal something that was repressed? Similar to ignorance, denial is a concept that tends to put the blame on people. What we suggest as an alternative avenue for interpretation is to move from a psychological concept to a philosophical concept. At stake is what Cora Diamond has called the ‘difficulty of reality’. By this, she means ‘the experience of the mind’s not being able to encompass something which it encounters. It is capable of making one go mad to try, to bring together in thought what cannot be thought’ [8]. The difficulty of reality concerns ‘experiences in which we take something in reality to be resistant to our thinking it, or possibility to be painful in its inexplicability’. This is a promising way of beginning to understand how cancer circulates as an object of knowledge in people’s lives. Our research has shown that for many cancer patients and family members in India, it is difficult to think about cancer: the disease is overwhelming, unaccountable and incomprehensible. It strikes out of the blue and is hard to get one’s mind around. It is often called a ‘big disease’. This ‘big disease’ is often addressed indirectly; people avoid even mentioning the word cancer. Whether it is deliberate hiding, or fear, or indifference is often difficult to say.

At stake, however, is not a lack of information or a lack of awareness. The difficulty is not related to things that people don’t know. It is related to things that people do know but wish they didn’t know and are afraid to say (and thus often hide or avoid or circumvent). Knowledge of cancer is knowledge that scares, knowledge that isolates and knowledge that wounds. The question is how people find a place in their lives for the things they happen to know but don’t want to know or would prefer not to know or don’t want to share.

What semi-structured interviews with open-ended questions bring to light is not necessarily what people really think about cancer, but the difficulty of thinking about cancer. Here, we see another limit of the survey: It might reveal what people think, but it won’t be able to reveal the difficulty of thinking. In the face of cancer, people tell incoherent stories. Why? Because people don’t simply know the disease, they are that knowledge. And that makes all the difference.

In the light of that difference, it might be worth pointing out another difference: Incoherence is a problem for the researcher; it is not a problem for the research participant. In fact, incoherence may be a response to the difficulty of reality, and, thus, a way of living with such a reality.

## References

[1] Weller D (2004). Behavioural and social science research in cancer: time for action. Eur J Cancer.

[2] Aapro MS, Aaro LE, Aro AR (2004). Research in the behavioural and social sciences to improve cancer control and care: a strategy for development. A report of an Expert Group. Eur J Cancer.

[3] Groves RM, Fowler FJ, Couper MP (2009). Survey Methodology.

[4] Bryman A (2016). Social Research Methods.

[5] Crano W, Brewer MB, Lac A (2014). Principles and Methods of Social Research.

[6] Sapsford R (2007). Survey Research.

[7] Vannette DL, Krosnick JA (2018). The Palgrave Handbook of Survey Research.

[8] Diamond C (2003). The difficulty of reality and the difficulty of philosophy *Partial Answers: Journal of Literature and the History of Ideas*.

